# Symbolic Number Ordering and its Underlying Strategies Examined Through Self-Reports

**DOI:** 10.5334/joc.157

**Published:** 2021-04-12

**Authors:** Natalia Dubinkina, Francesco Sella, Bert Reynvoet

**Affiliations:** 1 Brain and Cognition, KU Leuven, Belgium; 2 Faculty of Psychology and Educational Sciences, KU Leuven, Kulak, Kortrijk, Belgium; 3 Centre for Mathematical Cognition, Loughborough University, United Kingdom

**Keywords:** ordinality, numerical cognition, problem solving, mental arithmetic, strategic variations

## Abstract

Symbolic number ordering has been related to arithmetic fluency; however, the nature of this relation remains unclear. Here we investigate whether the implementation of strategies can explain the relation between number ordering and arithmetic fluency. In the first study, participants (N = 16) performed a symbolic number ordering task (i.e., “is a triplet of digits presented in order or not?”) and verbally reported the strategy they used after each trial. The analysis of the verbal responses led to the identification of three main strategies: memory retrieval, triplet decomposition, and arithmetic operation. All the remaining strategies were grouped in the fourth category “other”. In the second study, participants were presented with a description of the four strategies. Afterwards, they (N = 61) judged the order of triplets of digits as fast and as accurately as possible and, after each trial, they indicated the implemented strategy by selecting one of the four pre-determined strategies. Participants also completed a standardized test to assess their arithmetic fluency. Memory retrieval strategy was used more often for ordered trials than for non-ordered trials and more for consecutive than non-consecutive triplets. Reaction times on trials solved by memory retrieval were related to the participants’ arithmetic fluency score. For the first time, we provide evidence that the relation between symbolic number ordering and arithmetic fluency is related to faster execution of memory retrieval strategies.

## Introduction

Ordinality is a dimension of number processing that refers to the position of an item within a sequence (Gelman & Gallistel, 1978; Sury & Rubinsten, 2012). The processing of ordinality is typically assessed with a symbolic number ordering task in which participants indicate whether numbers of a triplet are in order or not (e.g., 1-2-3 vs 2-1-3; Lyons et al., 2016; Sury & Rubinsten, 2012). Number ordering has become a popular research topic in the domain of numerical cognition due to its relation with arithmetic fluency in children, from second grade onwards (Lyons, Price, Vaessen, Blomert, & Ansari, 2014; Sasanguie & Vos, 2018), and in adults (Sasanguie, Lyons, De Smedt, & Reynvoet, 2017). However, the cognitive mechanisms and strategies that support symbolic number ordering are still unclear (see Lyons, Vogel, & Ansari, 2016, for a review).

The manipulation of the numerical distance between digits (e.g., distance 1: 1-2-3; distance 2: 1-3-5) has led to some initial speculations on the underlying cognitive mechanisms of number order judgment. Individuals are faster in responding when digits in ordered triplets are consecutive (i.e., distance 1; 1-2-3) compared to non-consecutive ones (e.g., 1-4-7). This phenomenon is known as the reversed distance effect (RDE) as a small distance between digits in the triplets leads to faster reaction times (Goffin & Ansari, 2016; Lyons & Ansari, 2015; Lyons & Beilock, 2013; Sasanguie et al., 2017; Vogel et al, 2017; Vogel et al, 2019). Conversely, for not-ordered triplets (e.g. 3-1-2), individuals are slower in responding when triplets entail numbers with a small (e.g. 2-3-1) compared to large distance (e.g., 1-7-4). This difference is usually called standard distance effect (SDE; Morsanyi, O’Mahoney, & McCormack, 2017) as it mirrors the classic distance effect observed in digit comparison task (e.g., Moyer & Landauer, 1967; Buckley & Gillman, 1974), whereby reaction times are faster when comparing the magnitude of digits that are far apart (Moyer & Landauer, 1967).

These different behavioural signatures indicate that possibly different strategies are used for different types of trial in the symbolic number ordering task. Accordingly, it has been suggested that “magnitude-based” processes are used with not-ordered triplets, whereas “memory-based” mechanisms used with ordered triplets (Lyons et al., 2016; Sommerauer et al, 2020; Vos et al., 2017; Vogel et al, 2019). When presented with a not-ordered triplet, participants can only compare the magnitude of the individual digits? numbers? to arrive at a decision, resulting in a SDE as similarly observed in a number comparison task (Lyons & Beilock, 2013; Vos et al., 2017).

Different mechanisms could explain the presence of the RDE. Order judgment may rely on a serial visuospatial item-by-item scanning on a mental number line (Franklin et al., 2009; Turconi et al., 2004; Turconi et al., 2006). For triplets with consecutive digits (e.g., 1-2-3) only a small section of the number line needs to be scanned, whereas for triplets with larger between-item distances (e.g., 1-3-5), a larger section of the number line needs to be scanned. Alternatively, ordered triplets could be retrieved from long-term memory (Lefevre & Bisanz, 1986; Lyons & Beilock, 2013; Rubinsten et al., 2013; Vogel et al., 2017; Vos et al., 2017; Sella, Sasanguie & Reynvoet, 2020). In long-term memory, associative chains are formed between consecutive numbers. In these chains, each item triggers the next item in the sequence (e.g. 2 triggers 3, Serra & Nairne, 2000). Items that often co-occur will have stronger inter-item associations and, as a consequence, activate the next item more strongly. More specifically, consecutive triplets (i.e., 1-2-3) co-occur more frequently than triplets with a larger between-digit distance, such as 1-3-5, resulting in stronger associations and faster reaction times. Similarly, when the direction of the triplets is manipulated, faster reaction times are observed for ascending (e.g., 1-2-3) than descending (e.g., 3-2-1) trials (e.g., Vogel et al, 2019) presumably because ascending triplets co-occur more frequently, leading to stronger inter-item associations. These chaining mechanisms can also explain the observation that reaction times on consecutive triplets explain most of the individual variance in an arithmetic fluency test (Lyons et al., 2014). Arithmetic fluency is also a heavily memory-based skill (e.g., DeStefano & LeFevre, 2004; Hitch, 1978). The verification of correctly ordered consecutive sequences and arithmetic fluency possibly share the same process, that is, retrieving sequence knowledge (e.g., “1 2 3” and “2 times 3 is 6”; Sasanguie & Vos, 2018).

The precise contribution of different strategies in a symbolic order judgment task should be examined further, though. So far, evidence for the use of different strategies in the number order judgment task is indirectly inferred from reaction time differences between different types of trials (e.g., ascending vs descending, consecutive vs non-consecutive). Retrospective self-reports after every trial constitutes an effective methodology to assess strategy implementation. This method has been successfully applied to examine the use of strategies in mental arithmetic in children and adults (e.g., Torbeyns et al., 2016; 2019; Sella et al., 2019; see Lemaire & Siegler, 1995 for the method). Retrospective techniques might be well suited to gain more insight into the repertoire of strategies, their frequency and speed of execution in number ordering. In turn, the frequency and speed of execution of strategies can be related to arithmetic fluency. Specifically, it will be possible to determine whether arithmetic fluency correlates with the frequency and/or the speed of execution of certain solving strategies.

In the first study, we aimed to identify the common solution strategies that participants implement when judging the ordinality of triplets. Previous studies based on mental reports suggested that adults are capable of describing their solution processes retrospectively for simple arithmetic problems (LeFevre, Sadesky, & Bisanz, 1996). However, strategies for symbolic number ordering are, unlike arithmetic, not introduced as a part of the educational curriculum at schools. Therefore, we needed an exhaustive overview of possible strategies before assigning them to meaningful categories. In the second study, participants completed a number order judgment task as fast and as accurately as possible and, after each trial, indicated which strategy they used by selecting one among those categories derived from the first study. Additionally, in the second study, participants also completed an arithmetic fluency test as we aimed to get a better insight into strategies underlying symbolic order processing and its relation with arithmetic fluency.

## Study 1

### 1.1 Participants

Sixteen undergraduates from Psychology or Educational Sciences at KU Leuven Kulak (*M_age_* = 18.6; *SD* = 1.55; 14 female) took part in the study in exchange for course credits.

### 1.2 Procedure, task and stimuli

Participants received a booklet, whereby each page presented a triplet (font bold Courrier view, size 40). The task included 56 triplets; 28 ordered and 28 non-ordered, that were taken from the study of Vos et al. (2017). For 28 ordered triplets, there were 14 ascending and 14 descending. Half of the ascending and descending triplets consisted of consecutive triplets (e.g., 1-2-3 or 3-2-1), the other half of non-consecutive triplets with a numerical distance of 2, 3, or 4 (e.g., 1-4-7 or 7-4-1). Triplets with distance 2, 3 and 4 were grouped together to have an equal number of trials for consecutive and non-consecutive conditions. The distance between the first two digits and the last two digits of the triplet was always identical. The remaining 28 triplets were not-ordered and matched the size and the distance of the ordered ones (e.g. 1-3-2; 4-7-1). The full list of triplets is reported in *Appendix 1*.

Participants were instructed to choose between two options “correct” and “incorrect” and mark the right answer. After each triplet, they also needed to write down the strategy they implemented to decide whether the three digits were in order or not. They could choose between reporting their solutions in English or in Dutch (their first language) to avoid language-related difficulties in using math-related terminology. All the participants held nativelike proficiency in Dutch.

### 1.3. Results

The self-reports are available at OSF link. Two researchers (i.e., the first and last author) independently categorized participants’ self-reports into solution strategies. A solution strategy was labelled as “*Memory*” when participants rapidly recognized the presented triplet as being in order or not by matching it with stored representations in memory. Two examples of memory retrieval are “I immediately recognized the wrong order” or “I whispered the numbers in my head and they sounded right”. In “*Decomposition*”, participants conducted additional operations on the triplet, such as comparing two digits, replacing one digit with another digit within the sequence, adding a digit at the end or at the beginning, and reading the triplet backwards. Examples from this category are: “*7 is > 4 and 4 is < 1*”; “*There is no number possible between 1 (‘the first digit of the triplet’) and 2 (‘the last digit of the triplet’)*”; *“I looked for a pattern and then imaginary put 1 before the triplet (‘the original triplet was 234’)*”.

Based on the previous literature (Lefevre & Bisanz, 1986, Vos et al., 2017), we expected participants to implement memory retrieval and triplet decomposition as the two main solution strategies. However, not all reported strategies could be assigned to these two categories. A third strategy, “*Arithmetic*”, comprises the implementation of arithmetic procedures to determine the order of the triplet. For example, the arithmetical relation between the first two and last two digits was computed (e.g., triplet 5-3-4, that is first ‘-2’ and then ‘+1’) to decide whether the triplet was ordered or not. Other examples include digit division to make digits smaller to facilitate further recognition process. Ultimately, a small number of self-reported strategies with very heterogeneous nature could not be categorized in one of the three categories and were labelled as *“Other” strategies*. For example, “*I compared it with the trial before*” and “*I recognized odd numbers*”.

*To check the validity of the categorical system, a third independent rater, who was familiar with the task*,^1^ *was asked to assign the self-reports of half of the participants (N = 8; 447 trials)*^2^ *into one of the four categories. The inter-rater agreement was Cohen’s k = 0.81, which is considered as being high (Landis & Koch, 1977). The majority of the self-reports on which there was disagreement contained elements that could be linked with more than one category, such as, “I compared 1 with 2 and saw that 1 was added. And from 2 to 3 there is also 1 added. So I Simply recognized it” and “It is ascending from 3 to 5 with one. It’s also how we learn to count. I Simply recognized it*”.

**Figure 1:**
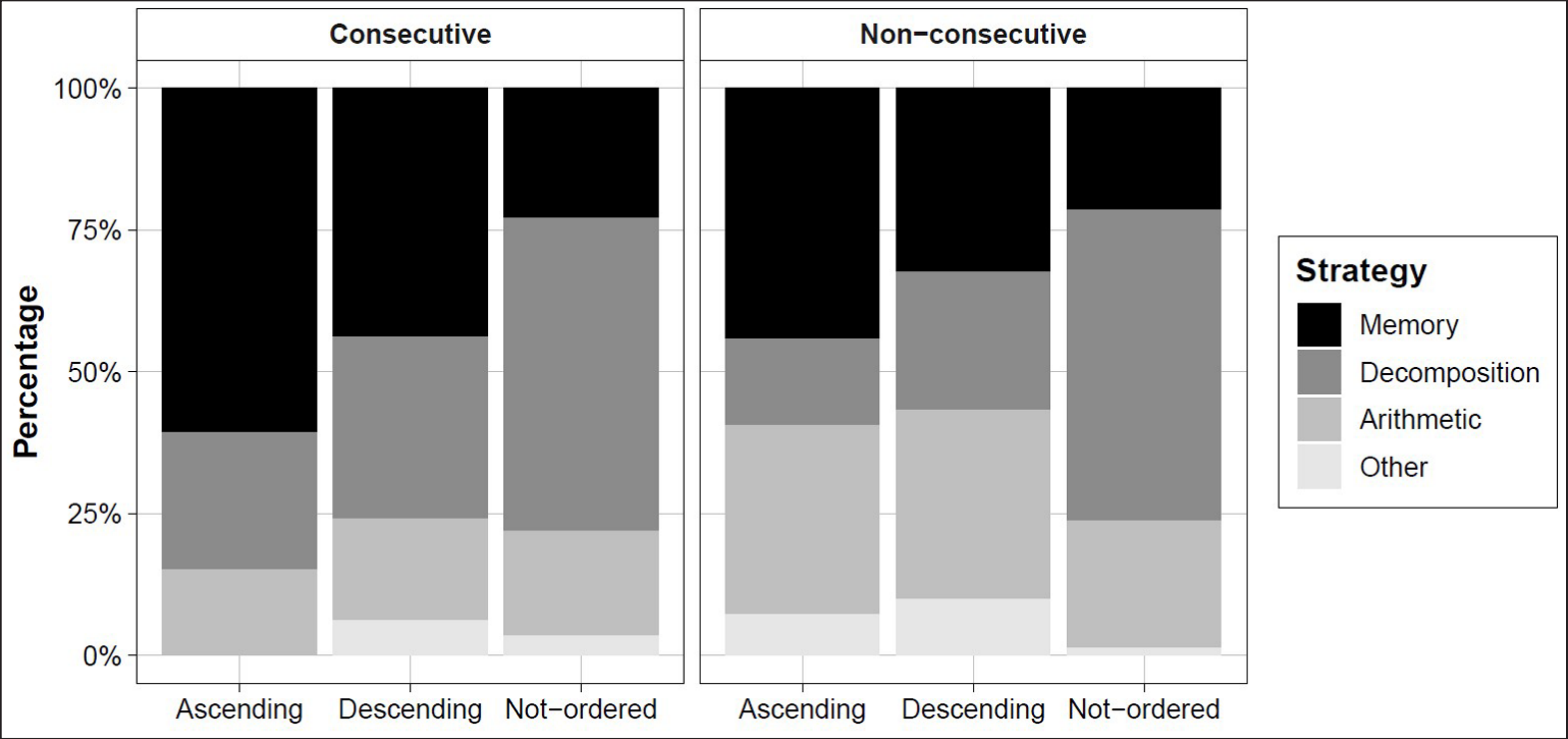
The percentage of strategy use (y-axis) as a function of the direction of the triplet (x-axis; ascending, descending, not-ordered) and the numerical distance between digits [Consecutive, left panel; Non-consecutive, right panel).

We then applied these categories to the dataset (N = 16; 894 trials)^3^ to calculate the use of the strategies for each of the conditions. We reported the percentage of strategies across conditions in ***Table 1*** and they are visually represented in ***Figure 1***.

**Table 1:** Distribution of the strategies (in percentage) per condition in Study 1 (AC: ascending consecutive; AnC: ascending non-consecutive; DC: descending consecutive; DnC: descending non-consecutive; NC: not-ordered consecutive; NnC: not-ordered non-consecutive).


	MEMORY (%)	DECOMPOSITION (%)	ARITHMETIC (%)	OTHER (%)

**AC**	60.71	24.11	15.18	0.00

**AnC**	44.14	15.32	33.33	7.21

**DC**	43.75	32.14	17.86	6.25

**DnC**	32.43	24.32	33.33	9.91

**NC**	22.77	55.36	18.30	3.57

**NnC**	21.43	54.91	22.32	1.34


***Figure 1*** and ***Table 1*** make clear that strategy use changed across conditions: The memory retrieval strategy was used more for ordered (than non-ordered), consecutive (than non-consecutive) and ascending (than descending) trials.

We analyzed the implementation of the memory strategy compared to the three remaining strategies, which were clustered together as “not-retrieval strategies”. We ran a logistic regression on memory strategy use [0 = not-memory, 1 = memory] with distance [consecutive, non-consecutive] and direction [ascending, descending, not-ordered] as predictors. We found the main effects of distance and direction (χ^2^ = 70.24, *p* < .001) whereas the interaction model was not significant (χ^2^ = 3.03, *p* = .22). Participants used more often memory retrieval in ascending compared to descending (Wald = 9.199, *p* = .002) and not-ordered triplets (Wald = 59.65, *p* < .001), and in consecutive than non-consecutive triplets (Wald = 5.88, *p* = .015).

### 1.4. Discussion

Study 1 aimed to identify the strategy repertoire in a symbolic number ordering task and examine the frequency of strategies across task conditions. We assigned participants’ solving strategies to four categories: memory retrieval, triplet decomposition, arithmetic operations and “other” strategies. Participants used memory retrieval more often for ascending triplets compared to descending and not-ordered ones, and for consecutive compared to non-consecutive digits. This result suggests the use of memory retrieval, either through verbal or visual recognition, when triplets matched a portion of the counting list (Lyons & Beilock, 2013; Rubinsten et al., 2013; Vogel et al., 2017; Vos et al., 2017).

Decomposition, which entails the sequential magnitude comparison of digits within the sequence, was most widely used for not-ordered triplets. We speculate that participants adopted a decomposition strategy when they realized that the triplet did not belong to the counting list (forward or backwards). The strategy based on arithmetic operations was not previously contemplated (see Lefevre & Bisanz, 1986), but it was used quite often (about 22.6% of the total amount of the trials), especially in ascending and descending trials with large distances (i.e., non-consecutive digits). Again, when the digits in the sequence were not consecutive, participants resorted to procedural, rather than memory retrieval, strategies.

In summary, by assessing retrospective self-reports, Study 1 demonstrated that participants use a variety of solution strategies in a symbolic number ordering task. We propose the presence of three main strategies: memory retrieval, decomposition, and arithmetic operations. Descriptive statistics showed that memory retrieval is used more often for ordered triplets with consecutive digits, whereby a direct recognition that the triplet belongs to the counting list can be made. Conversely, not-ordered triplets with non-consecutive digits prompted the use of more sequential strategies, such as comparing the magnitude of digits or performing some arithmetical operations.

In Study 2, we aimed at replicating these results in a larger sample, using a choice menu for assessing strategies instead of retrospective self-reports. We also registered reaction times to analyse the speed of execution for each strategy. Finally, we measured participants’ arithmetic fluency to evaluate whether the frequency and the speed of execution of one or more strategies is related to arithmetic skills.

## Study 2

### 2.1. Participants

Sixty-one undergraduates from Applied Economics at KU Leuven Kulak (*M_age_* = 18.22; *SD* = 0.72; 27 female) took part in this study in exchange for course credits.

### 2.2. Procedure, task and stimuli

At the beginning of the experimental session, participants completed the Tempo Test Rekenen (TTR; Vos, 1992). The TTR is a time-limited test consisting of five columns on a sheet of paper: addition, subtraction, multiplication, division and one with mixed operations. Each column consists of forty arithmetic problems (e.g., 12 × 3 = ___) presented in increasing difficulty. Participants completed one column at time and had one minute to solve as many problems as possible in a column. We calculated the total number of correct responses.

After completing the arithmetic fluency test, participants were introduced to the number order judgement task and the strategies derived from Study 1. They received sheets with explanations of the four strategies and some examples (The description of the strategies can be found at OSF link). The symbolic number ordering task was presented via E-prime 2.0 (Psychological Software Tools, Pittsburgh, PA, USA). Participants indicated whether three digits presented in the middle of the screen (res. 1920x1080) were ordered (i.e., ascending or descending), or not-ordered by pressing the ‘a’ or ‘p’ key on an AZERTY keyboard, respectively. After responding, participants reported which strategy they employed by choosing among the four strategies derived from Study 1, namely, memory, decomposition, arithmetic and “other” by pressing the keys ‘a’, ‘b’, ‘c’, and ‘d’, respectively. There was no time limit for selecting the used strategy. Fifteen practice trials were presented to make participants familiar with the procedure. Then, the same 56 triplets used in Study 1 were presented randomly. Accuracy and reaction times, together with the strategy choices, were recorded. The dataset is available at OSF link.

### 2.3. Results

We removed responses below 200 msec (i.e., anticipations; 6 trials) and extremely slow responses (44 trials) that were three standard deviations above the grand mean. We then calculated the individual median reaction times of correct responses. We removed one participant from further analysis because had a median response time that was more than three standard deviations above the sample mean. Accuracy was close to ceiling (*M* = 0.94, *SD* = 0.05) and therefore not analysed.

First, we examined the presence of reversed distance effect (RDE) and distance effect (DE) in the response times (RTs) across conditions. Second, we evaluated the frequencies of strategies and execution times of memory compared to the remaining strategies. Finally, we examined the relation between the performance in the order judgement task and arithmetic fluency.

## Distance effects

To check to which extent our data replicated behavioral effects from previous studies (Franklin et al., 2009; Goffin & Ansari, 2016; Morsanyi et al., 2017; Vogel et al., 2017; Vos et al., 2017), we analyzed the median reaction times of correct responses in a repeated-measures ANOVA with direction [Ascending, Descending, Not-ordered] and distance [Consecutive, Non-consecutive] as within-subjects factors. Whenever the assumption of sphericity was violated, the Greenhouse–Geisser correction was applied. The main effect of distance was not significant, *F*(1, 59) = 2.66, *p* = .108, η^2^_g_ = .003, whereas the main effect of direction was, *F*(2, 118) = 7.42, *p*_[gg]_ = .002, η^2^_g_ = .02. Crucially, the interaction between distance and direction was also significant, *F*(2, 118) = 9.51, *p* < .001, η^2^_g_ = .01. Participants were faster with consecutive (*M* = 1377, *SD* = 452) compared to non-consecutive digits (*M* = 1501, *SD* = 490) in ascending triplets, that is the RDE, *t*(59) = 2.93, *p*_[bonf]_ = .015. A similar tendency for a RDE was observed for descending triplets (consecutive: *M* = 1514, *SD* = 504; non-consecutive: *M* = 1625, *SD* = 568; *t*(59) = 2.31, *p*_[bonf]_ = .075). No such difference was observed between consecutive and non-consecutive triplets in the not-ordered trials (consecutive: *M* = 1622, *SD* = 461; non-consecutive: *M* = 1540, *SD* = 478; *t*(59) = 2.03, *p*_[bonf]_ = .141).

### Strategy use

**Figure 2:**
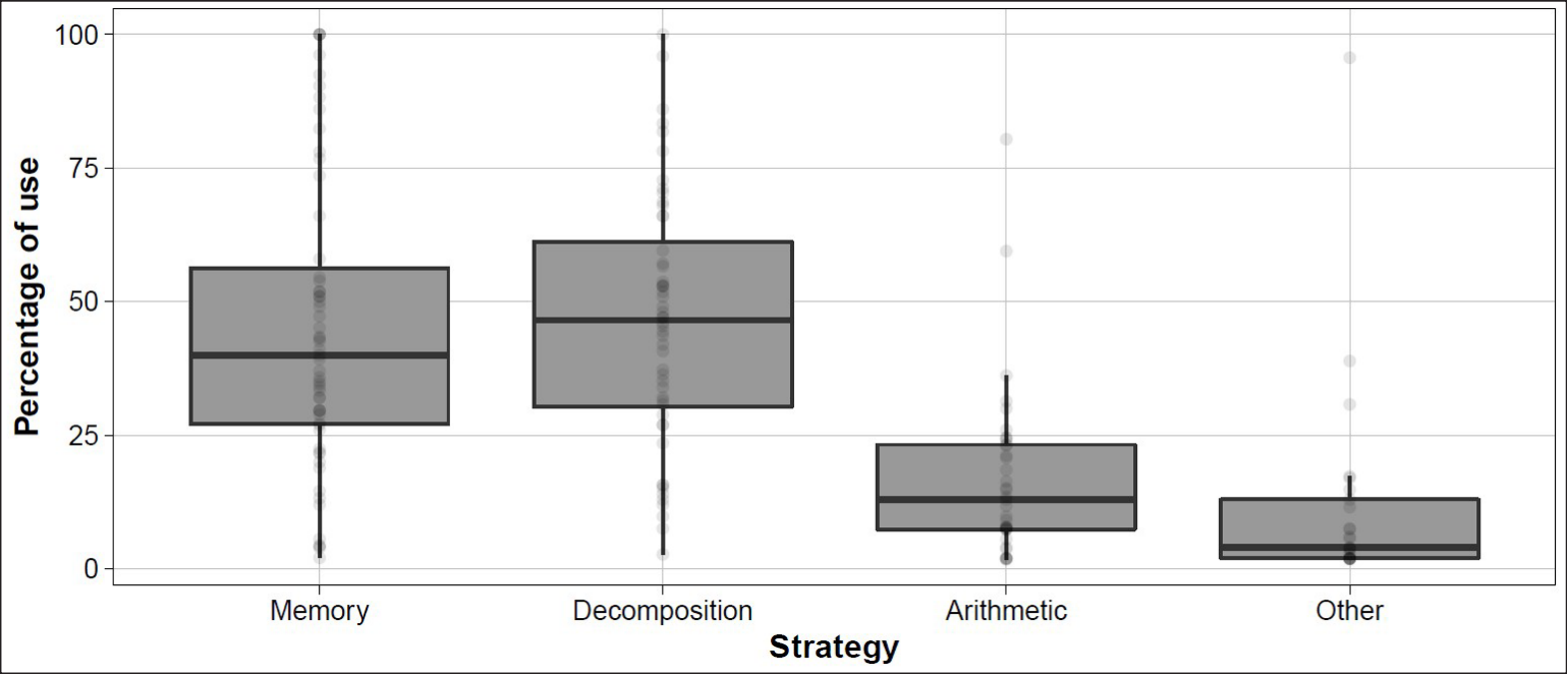
Boxplots represent the distribution of percentages of strategy use (y-axis); dots represent individual values.

In ***Figure 2***, we reported the distributions of use (in percentage) of each strategy. Memory and decomposition were two widely used strategies, whereas arithmetic and “other” strategies were applied less often. One participant (1.67% of the sample) never used memory, 8 participants (13.33%) never used decomposition, 23 participants (38.33%) never used arithmetic, and 35 participants (58.33%) never reported “other” strategies.

**Figure 3:**
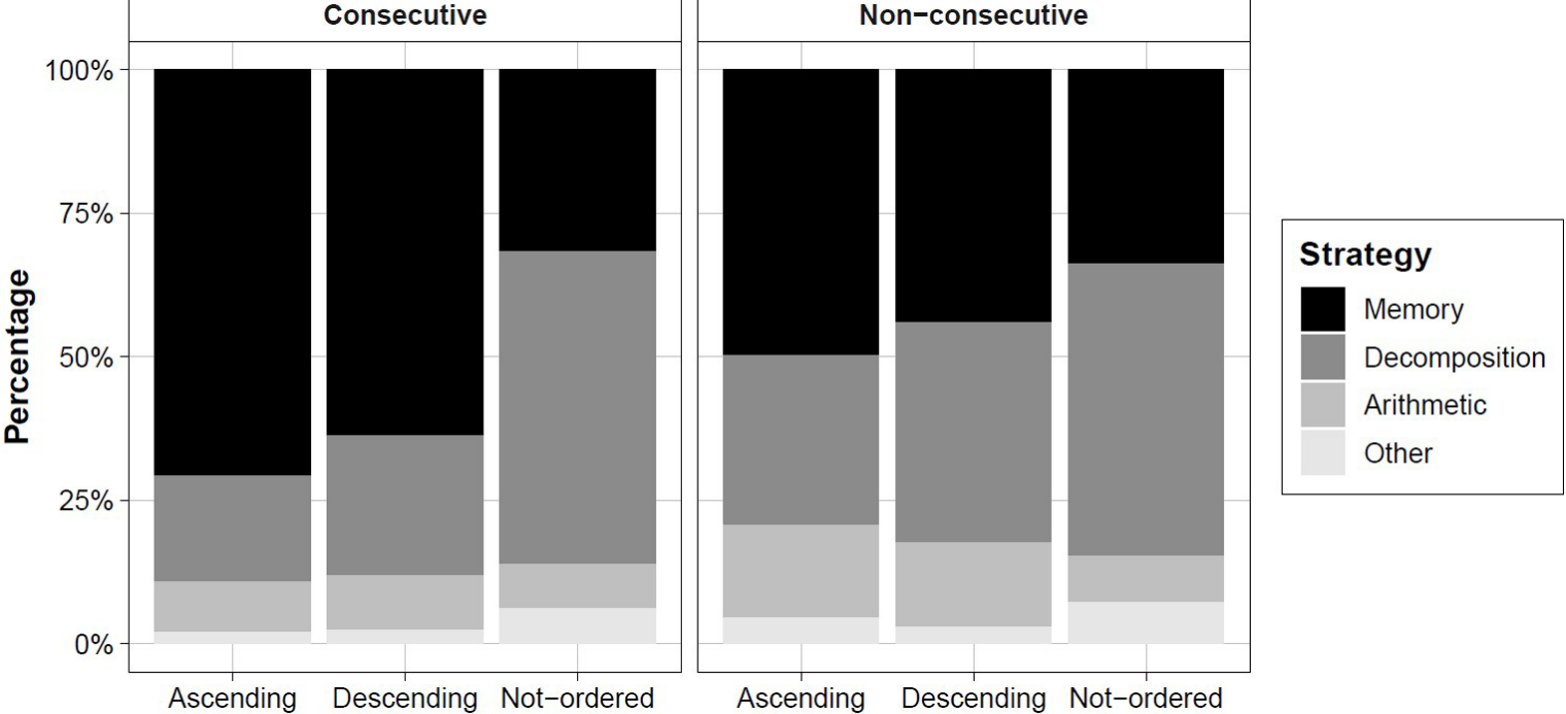
The percentage of strategy use (y-axis) as a function of the direction of the triplet (x-axis; ascending, descending, not-ordered) and the numerical distance between digits [Consecutive, left panel; Non-consecutive, right panel).

In ***Figure 3***, we plotted the percentages of the strategies (i.e., memory, decomposition, arithmetic, other) across the six conditions (i.e., ascending, descending, not-ordered triplets with consecutive or non-consecutive digits) of the number order judgment task. The percentages can be also found in ***Table 2***.

**Table 2:** Distribution of the strategies (in percentage) per condition in Study 2 (AC: ascending consecutive; AnC: ascending non-consecutive; DC: descending consecutive; DnC: descending non-consecutive; NC: not-ordered consecutive; NnC: not-ordered non-consecutive).


	MEMORY (%)	DECOMPOSITION (%)	ARITHMETIC (%)	OTHER (%)

**AC**	70.68	18.55	8.77	2.01

**AnC**	49.74	29.64	15.98	4.64

**DC**	63.73	42.35	9.59	2.38

**DnC**	44.09	38.22	14.70	2.89

**NC**	31.66	54.44	7.72	6.18

**NnC**	33.84	50.88	8.08	7.20


We analyzed the implementation of the memory strategy compared to the three remaining strategies, which were clustered together as “not-retrieval strategies”. We ran a logistic regression on memory strategy [0 = not-memory, 1 = memory] use with distance [consecutive, non-consecutive] and direction [ascending, descending, not-ordered] as predictors. We found evidence for the interaction model compared to the model with the two main effects (χ^2^ = 39.81, *p* < .001). When triplets were consecutive, participants used more often memory retrieval for ascending compared to descending (Wald = 4.29, *p* = .038) and not-ordered triplets (Wald = 150.75, *p* < .001). When triplets were non-consecutive, participants used more often memory retrieval for ascending compared to not-ordered trials (Wald = 27.33, *p* < .001), but not for descending triplets (Wald = 2.46, *p* = .117).

**Figure 4:**
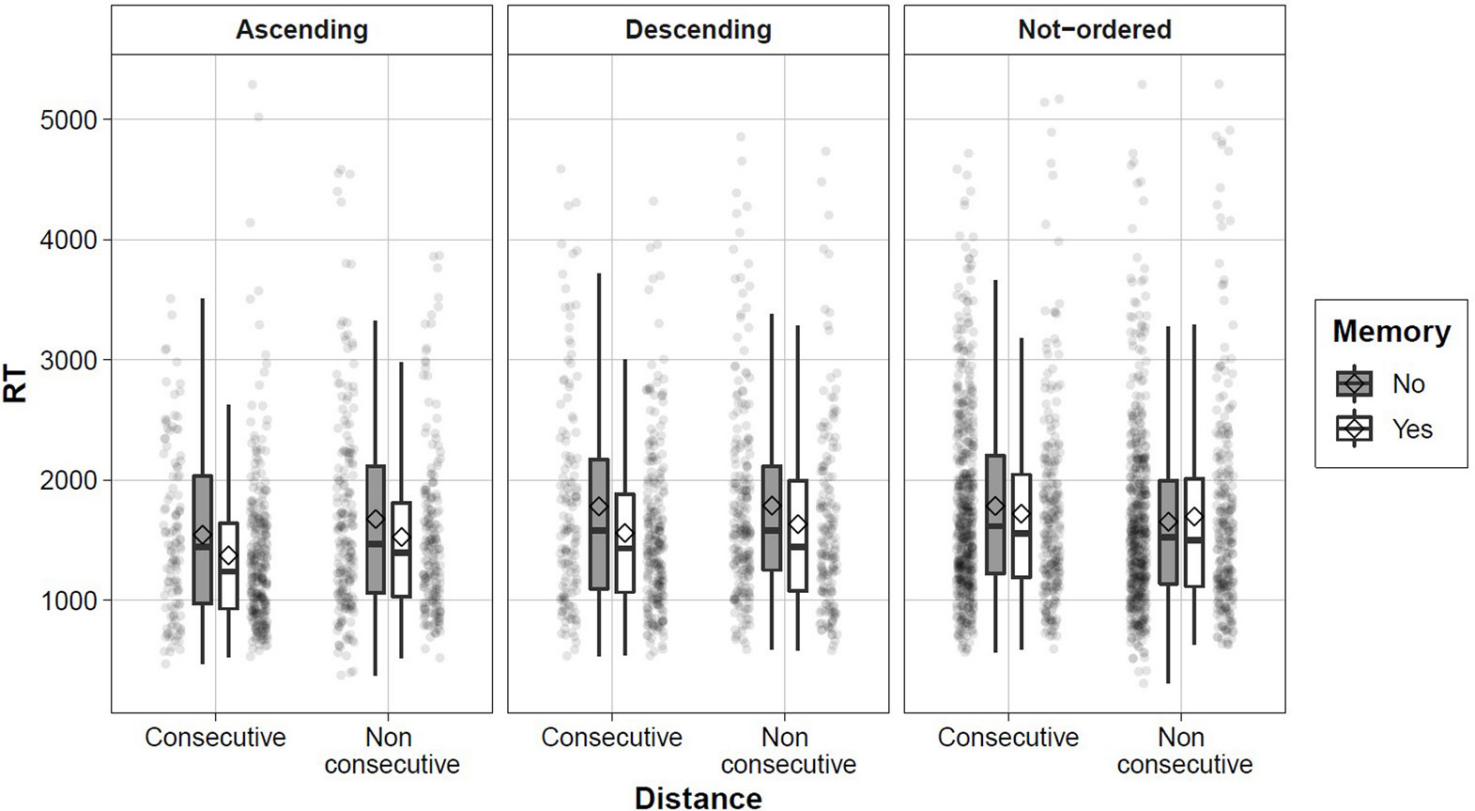
Boxplots of reaction times (y-axis) for trials in which the memory strategy was used (Yes; white) or not (No; dark grey) across direction (left: Ascending; middle: Descending; right: Not-ordered) and distance (x-axis). The diamonds represent the mean of the distribution whereas transparent dots represent single trials.

We also analyzed the execution times of the different strategies. The implementation of the memory strategy, which is based on the immediate retrieval, should yield faster reaction times compared to the remaining non-memory strategies, which, by definition, require a series of additional procedures or manipulations in order to verify whether a triplet was in order or not. In ***Figure 4***, we plotted the distributions of reaction times for trials in which participants reported using the memory strategy compared to those in which remaining strategies were used across the conditions of the number order judgment task. Participants were indeed faster when they reported using a memory strategy (*M* = 1431, *SD* = 394) compared to other non-memory strategies (*M* = 1615, *SD* = 457; *t*(54) = –4.31, *p* < .001; five participants were excluded because they did not use both strategies).

## The relation between symbolic number ordering and arithmetic fluency

We examined the zero-order correlations between arithmetic fluency and the overall median RT and the median RTs for each condition separately (***Table 3***). The RTs in the descending consecutive and non-consecutive conditions yielded the highest correlations with arithmetic fluency, even though there is no significant difference (Z = 1.27, *p* = .21; Steiger, 1980; Diedenhofen & Musch, 2015) between the smallest and the largest correlation observed in one of the ordered conditions and TTR (“ascending non-consecutive”: *r* = –0.36 vs. “descending non-consecutive”: *r* = –0.44). Correlations between RTs on non-ordered trials and arithmetic fluency were not significant. The correlation between median RTs on all ordered trials (i.e., ascending consecutive, ascending non-consecutive, descending consecutive, and descending non-consecutive) and TTR was significant (*r* = -.53, *p* < .001), after controlling for the median RTs on all non-ordered trials.

**Table 3:** Zero-order correlations between overall median RTs (All), median RTs across conditions (AC: ascending consecutive; AnC: ascending non-consecutive; DC: descending consecutive; DnC: descending non-consecutive; NC: not-ordered consecutive; NnC: not-ordered non-consecutive), and arithmetic fluency score (TTR). ** *p* < .01.


MEASURE	1	2	3	4	5	6	7

1. All							

2. AC	.79**						

3. AnC	.88**	.76**					

4. DC	.86**	.75**	.72**				

5. DnC	.85**	.71**	.85**	.76**			

6. NC	.88**	.59**	.70**	.69**	.63**		

7. NnC	.85**	.54**	.72**	.59**	.65**	.78**	

8. TTR	–.35**	–.37**	–.36**	–.44**	–.44**	–.12	–.19


To get a better insight into the relation between the frequency and efficiency of strategy use, we correlated the proportion of the memory strategy use and median RTs for memory retrieval and decomposition strategies with the TTR scores (***Table 4***). We excluded 9 participants who never used either memory or decomposition strategy. We did not compute the correlation between RTs for the arithmetic and “other” strategies and TTR because several participants never used these strategies (see ***Figure 4***). The frequency of the memory retrieval strategy was not related to arithmetic fluency, indicating that memory retrieval was not used more often by participants with a high arithmetic fluency. The mean execution time of the memory strategy and the score on the TTR were significantly correlated (*r* = -.31), indicating that participants who executed the retrieval strategy in the ordering task faster, performed better on the arithmetic fluency test. The correlation between the RTs of the memory strategy and TTR was not significantly different from the correlation between the RTs from the decomposition strategy and TTR (Z = –1.64, *p* = .10).

**Table 4:** Zero-order correlations between proportion of memory strategy use, median RTs for memory, median RTs for decomposition, and arithmetic fluency (TTR). * *p* < .05, ** *p* < .01.


MEASURE	1	2	3

1. Proportion memory strategy			

2. Median RTs for memory strategy	–.04		

3. Median RTs for decomposition strategy	.12	.67**	

4. TTR	.13	–.31*	–.12


## Discussion

The aim of Study 2 was twofold. First, we examined whether the findings from Study 1 could be replicated in a larger sample by assessing strategies with a retrospective choice menu instead of free self-reports. All categories of strategies derived on the basis of free retrospective reports in Study 1 were selected on several occasions. In general, the memory retrieval strategy (45%) and the decomposition strategy (40%) were reported more often. The arithmetic operations strategy was reported in only 10% of the trials. This pattern is similar to the one observed in Study 1. Moreover, as in Study 1, the frequency of each strategy varied across conditions: memory retrieval was used more in ordered conditions, especially with consecutive triplets (i.e., consecutive triplets; ***Figure 3***). For non-ordered trials, the decomposition strategy was reported as the most frequently used. Importantly, the retrospective reports of the participants made clear that all three strategies were applied in all conditions of the symbolic number order task. Thus, our data shows that reaction times observed in each condition are always a combination of execution times of different strategies.

A second aim of Study 2 was to examine whether frequency and efficiency of strategies related to arithmetic fluency. The frequency of the memory retrieval strategy was not related to arithmetic fluency. In contrast, participants doing well on the arithmetic fluency task were faster on number ordering trials in which they reported using memory retrieval. We return to the relevance of this finding in our general discussion.

### General Discussion

Recently, a lot of research has been devoted to the symbolic number ordering task judgement because of its relation with arithmetic fluency (for reviews see Lyons, Vogel, & Ansari, 2016; Sury & Rubinsten, 2011). However, the shared cognitive processes responsible for this relationship remain unclear. In this paper, we explored the cognitive processes involved in number ordering, by registering, for the first time, trial-by-trial retrospective reports on strategy use. In this way, we gained insights in the different strategies and why number order relates to arithmetic fluency.

The first aim of this study was to identify the strategies applied in the symbolic number order judgment task. In contrast to the field of arithmetic, in which strategies are introduced as a part of the educational curriculum at schools, we needed to define a repertoire of strategies in number ordering. Therefore, in Study 1, we analyzed participants’ self-reports on solving strategies. We identified three main strategies: memory retrieval, triplet decomposition, and arithmetic operations. A few self-reports did not fit in one of these three categories and were labelled as “*other*” strategies. In Study 2, participants completed a number order judgment task and, after each trial, they chose the strategy they implemented among one of the four strategies identified in Study 1. Both studies revealed that memory retrieval and decomposition strategies were used most frequently. Strategies relying on arithmetic operations were used less often. Only about 4–5% of the applied strategies did not fit into one of these categories.

Memory retrieval was more frequently used for ordered triplets, especially with consecutive digits. In contrast, decomposition was applied more often for non-ordered triplets. Presumably, memory strategies are more often used when the triplet matches the counting list either forward or backwards. When the triplet does not match the counting list, participants used more sequential strategies like decomposition. It remains an open question whether participants compare the triplet with the counting list and, in case of a non-match, apply decomposition or arithmetic strategies or whether the strategies are run in parallel.

Our findings are in line with previous studies (Vos et al., 2017; Sella et al., 2020), in which a similar proposal was made for different solution strategies usage for ordered and not-ordered triplets. This assumption was based on reaction times that displayed a reversed and a standard distance effect in ordered and non-ordered conditions respectively. This reaction time pattern was replicated in Study 2: the distance effect interacted with condition (ascending, descending, not-ordered). We should note also that the standard distance effect in the not-ordered condition was in the expected direction, but not statistically significant. Possibly, this is caused by more noise in the reaction times, either caused by a reduction of trials compared with previous studies (we only presented each triplet once in this study), or alternatively, due to the dual-task requirement (i.e., verifying order and reporting strategy).

As expected, response times were faster when memory retrieval strategy was applied compared to non-memory retrieval strategies. This has important consequences for the origin of the reversed distance effect. Previous studies argued that the reversed distance may be the consequence of different association strengths between consecutive and non-consecutive digits (Franklin et al, 2009; Lyons & Beilock, 2013; Vogel et al., 2017; Vogel et al., 2019; Vos et al., 2017). More specifically, consecutive triplets (e.g., 1-2-3) co-occur more frequently than triplets with a larger between-digit distance, such as 1-3-5, resulting in stronger associations and faster retrieval for consecutive triplets (Vos et al., 2017). Study 2 provides supportive evidence for this account: the frequency of memory retrieval is higher for ordered triplets with consecutive digits than for ordered triplets with non-consecutive digits (see ***Figure 3***). Because memory retrieval is faster than other strategies, a logical consequence is that average reaction times in the ordered consecutive triplets will be faster than in the ordered non-consecutive triplets, that is the reversed distance effect. A similar explanation may hold for the direction effect previously reported, that is, faster reaction times on ascending than descending trials (Vos et al. 2017; Vogel et al, 2019): memory retrieval is reported more for ascending trials, which could give rise to the direction effect. The present observation makes clear that using the average reaction time data per condition can provide a misleading picture on the behavioral patterns and underlying cognitive processes in the symbolic number ordering task, because the frequency of strategies differs across conditions.

The second aim was to get a better insight into the association between symbolic number ordering and arithmetic proficiency. In line with previous studies, we found that fast response times in the order judgment task are associated with better arithmetic fluency (Sasanguie et al, 2017; Lyons & Beilock, 2011, 2013; Lyons et al., 2014; Morsanyi, Mahoney, & Mccormack, 2016; Morsanyi, van Bers, O’Connor, & McCormack, 2018; Rubinsten & Sury, 2011; Sasanguie & Vos, 2018; Vogel et al., 2017; Vogel, Remark, & Ansari, 2014; Vos et al., 2017). When analyzing the task conditions, we found the reaction times in descending non-consecutive trials had the strongest correlation with arithmetic fluency, although the correlation was not significantly larger than the lowest observed among ascending conditions (i.e., ascending non-consecutive). In contrast, reaction times in not-ordered trials were not related to arithmetic (***Table 3***). This observation is at first sight somewhat different than the one in Lyons & Ansari (2015), who found that the biggest variance in arithmetic was explained by the performance on consecutive ascending triplets (i.e., 1-2-3). However, it is difficult to compare both studies as Lyons and Ansari, in contrast to this study, studied primary school children and did not present descending trials in their design. The fact that the largest correlations between symbolic number ordering and arithmetic fluency was observed for conditions in which proportionally more retrieval was used (ascending/descending ordered conditions) suggests that memory retrieval is the common process responsible for the relation. This was confirmed by the fact that only RTs on trials where retrieval was used were related to arithmetic fluency (***Table 4***). This is in line with the proposal of Sasanguie and Vos (2018), who argued that both the symbolic number ordering task and an arithmetic fluency task require memory retrieval (see also Sella et al., 2020). Moreover, a recent neuroimaging study of Sommerauer and colleagues (Sommerauer, Grass, Grabner, & Vogel, 2020) also suggests that children with high arithmetic performance complete symbolic ordering task faster than less proficient control group due to more efficient retrieval of sematic associations, stored in long-term memory. Next to more efficient memory retrieval, we also aimed to verify whether arithmetic proficiency influenced the frequency of the memory retrieval strategy. This was not the case: more arithmetic proficient participants did not use this strategy more than less skilled ones.

The findings of the present study should be considered in light of some limitations. First, the distance between the first two and last two digits of each ordered triplet was always the same. The regular pattern of the ordered triplets may have triggered the memory retrieval strategy, resulting in an overestimation of this strategy in the current study. Further studies are needed to examine whether including triplets with varying inter-item distances (e.g., 2-4-7) influences the frequency of strategies.

Second, the homogeneity of our sample may have resulted in participants using the different strategies with similar frequency. Therefore, whether arithmetically skilled and less-skilled participants differ present a different frequency distribution of strategies remains to be further investigated in a more heterogeneous sample. Related, our findings cannot be generalized to children, who are still learning associations between single digits (i.e., ordering) and associations between arithmetic problems and solutions (i.e., arithmetic fluency).

The final limitation concerns the validity of the retrospective self-reports as an empirical method. For instance, participants can differ with respect to the ability to give these retrospective reports; there is no consistent evidence yet for a relation between the awareness level of the strategy usage and its further verbalization (for the discussion, see Lemaire, 2016), and it could be the case that some participants are more confident in reporting their solutions retrospectively and that their reports are a more reliable source of information. There are also no reliable ways to distinguish between mental operations that can be easily verbalized and those which are more difficult to verbalize. However, some work has shown that verbal reports are a valid source of data (Robinson, 2001) and can provide a more accurate representation of the applied strategies than accuracy and reaction times in a variety of mental arithmetic tasks (Siegler, 1989; Siegler & Stern, 1998). The validity of self-reports is also supported by neuroscientific evidence (Grabner & De Smedt, 2011; Grabner, Brunner, Lorenz, Vogel, & De Smedt, 2020). For instance, Grabner and De Smedt (2011) observed different oscillatory brain responses for arithmetic problems for which participants reported to have used retrieval compared to arithmetic procedures. In future studies, the validity of self-reports in the symbolic order judgement task could be further investigated with eye movements. Accordingly, eye movements can reveal the implementation of strategies as demonstrated in other numerical tasks (e.g., Van’t Noordende, Van Hoogmoed, Schot & Kroesbergen, 2016; Paul, Reeve, & Forte, 2020). In symbolic number ordering, eye movements may go back and forth between the digits when a decomposition strategy is used whereas fewer movements may be performed in the case of memory retrieval.

## Conclusions

Using retrospective self-reports, this study demonstrated the distribution of strategies across different conditions of the symbolic number ordering task. Memory retrieval was used more often for ordered sequences and decomposition for not-ordered sequences. The well-established relation between symbolic number ordering and arithmetic fluency is due to faster execution of memory retrieval strategies, suggesting that the common core between symbolic number ordering and arithmetic is memory retrieval.

## Data Accessibility Statements

OSF project link: *https://osf.io/hypcr/?view_only=b95fd149f71645d28999caaf63b300ce*.

## Additional File

The additional file for this article can be found as follows:

10.5334/joc.157.s1 Appendix 1. Stimulus list.

## Footnotes

Helene Vos. PhD in Psychology.

One observation was missed due to a participant skipping a trial.

Two observations were missing because two participants skipped a trial.

## References

[1] Buckley, P. B., & Gillman, C. B. (1974). Comparisons of digits and dot patterns. Journal of Experimental Psychology, 103(6), 1131–1136. DOI: 10.1037/h0037361 4457588

[2] DeStefano, D., & LeFevre, J.-A. (2004). The role of working memory in mental arithmetic. European Journal of Cognitive Psychology, 16(3), 353–386. DOI: 10.1080/09541440244000328

[3] Diedenhofen, B., & Musch, J. (2015). cocor: A Comprehensive Solution for the Statistical Comparison of Correlations. PLOS ONE, 10(4), e0121945. DOI: 10.1371/journal.pone.0121945 25835001 PMC4383486

[4] Franklin, M. S., Jonides, J., & Smith, E. E. (2009). Processing of order information for numbers and months. Memory & Cognition, 37(5), 644–654. DOI: 10.3758/MC.37.5.644 19487756

[5] Gelman, R., & Gallistel, C. R. (1978). The child’s understanding of number. Washington, DC: American Psychological Association.

[6] Goffin, C., & Ansari, D. (2016). Beyond magnitude: Judging ordinality of symbolic number is unrelated to magnitude comparison and independently relates to individual differences in arithmetic. Cognition, 150, 68–76. DOI: 10.1016/j.cognition.2016.01.018 26851638

[7] Hitch, G. J. (1978). The role of short-term working memory in mental arithmetic. Cognitive Psychology, 10(3), 302–323. DOI: 10.1016/0010-0285(78)90002-6

[8] Landis, J., & Koch, G. (1977). The measurement of observer agreement for categorical data. Biometrics, 33, 159–174. DOI: 10.2307/2529310 843571

[9] LeFevre, J.-A., & Bisanz, J. (1986). A cognitive analysis of number series problems: Sources of individual differences in performance. Memory & Cognition, 14(4), 287–298. DOI: 10.3758/BF03202506 3762382

[10] LeFevre, J.-A., Sadesky, G. S., & Bisanz, J. (1996). Selection of procedures in mental addition: Reassessing the problem size effect in adults. Journal of Experimental Psychology: Learning, Memory, and Cognition, 22(1), 216–230. DOI: 10.1037/0278-7393.22.1.216

[11] Lemaire, P. (2016). Cognitive aging: The role of strategies. Psychology Press. DOI: 10.4324/9781315650999

[12] Lemaire, P., & Siegler, R. S. (1995). Four aspects of strategic change: Contributions to children’s learning of multiplication. Journal of Experimental Psychology: General, 124(1), 83–97. DOI: 10.1037/0096-3445.124.1.83 7897342

[13] Lyons, I. M., & Beilock, S. L. (2013). Ordinality and the nature of symbolic numbers. Journal of Neuroscience, 33(43), 17052–17061. DOI: 10.1523/JNEUROSCI.1775-13.2013 24155309 PMC6618433

[14] Lyons, I. M., Price, G. R., Vaessen, A., Blomert, L., & Ansari, D. (2014). Numerical predictors of arithmetic success in grades 1–6. Developmental Science, 17(5), 714–726. DOI: 10.1111/desc.12152 24581004

[15] Lyons, I. M., Vogel, S. E., & Ansari, D. (2016). On the ordinality of numbers: A review of neural and behavioral studies. Progress in Brain Research, 227, 187–221. DOI: 10.1016/bs.pbr.2016.04.010 27339013

[16] Morsanyi, K., O’Mahoney, E., & McCormack, T. (2016). Number comparison and number ordering as predictors of arithmetic performance in adults: Exploring the link between the two skills, and investigating the question of domain- specificity. Quarterly Journal of Experimental Psychology, 70, 2497–2517. DOI: 10.1080/17470218.2016.1246577 27734751

[17] Morsanyi, K., van Bers, B. M. C. W., O’Connor, P., & McCormack, T. (2018). Developmental Dyscalculia is Characterized by Order Processing Deficits: Evidence from Numerical and Non-Numerical Ordering Tasks. Developmental Neuropsychology, 43(7), 595–621. DOI: 10.1080/87565641.2018.1502294 30058838

[18] Moyer, R. S., & Landauer, T. K. (1967). Time required for judgements of numerical inequality. Nature, 215, 1519–1520. DOI: 10.1038/2151519a0 6052760

[19] Paul, J., Reeve, R. A., & Forte, J. A. (2020). Enumeration strategy differences revealed by saccade-terminated eye tracking. Cognition, 198(1), 104204. DOI: 10.1016/j.cognition.2020.104204 32014714

[20] Robinson, K. M. (2001). The validity of verbal reports in children’s subtraction. Journal of Educational Psychology, 93(1), 211–222. DOI: 10.1037/0022-0663.93.1.211

[21] Rubinsten, O., Dana, S., Lavro, D., & Berger, A. (2013). Processing ordinality and quantity: ERP evidence of separate mechanisms. Brain and Cognition, 82(2), 201–212. DOI: 10.1016/j.bandc.2013.04.008 23681053

[22] Rubinsten, O., & Sury, D. (2011). Processing ordinality and quantity: The case of developmental dyscalculia. PLoS ONE, 6(9). DOI: 10.1371/journal.pone.0024079 PMC3174157 21935374

[23] Sasanguie, D., Lyons, I., De Smedt, B., & Reynvoet, B. (2017). Unpacking symbolic number comparison and its relation with arithmetic in adults. Cognition, 165, 26–38. DOI: 10.1016/j.cognition.2017.04.007 28460351

[24] Sasanguie, D., & Vos, H. (2018). About why there is a shift from cardinal to ordinal processing in the association with arithmetic between first and second grades. Developmental Science, 21(5), 1–13. DOI: 10.1111/desc.12653 29417697

[25] Sella, F., Re, A. M., Lucangeli, D., Cornoldi, C., & Lemaire, P. (2019). Strategy Selection in ADHD Characteristics Children: A Study in Arithmetic. Journal of Attention Disorders, 23(1), 87–98. DOI: 10.1177/1087054712438766 22451509

[26] Sella, F., Sasanguie, D., & Reynvoet, B. (2020). Judging the order of numbers relies on familiarity rather than activating the mental number line. Acta Psychologica, 204(6), 103014. DOI: 10.1016/j.actpsy.2020.103014 32004925

[27] Serra, M., & Nairne, J. S. (2000). Part-set cuing of order information: implications for associative theories of serial order memory. Memory & Cognition, 28(5), 847–855. DOI: 10.3758/BF03198420 10983459

[28] Siegler, R. S. (1989). Hazards of mental chronometry: An example from children’s subtraction. Journal of Educational Psychology, 81(4), 497–506. DOI: 10.1037/0022-0663.81.4.497

[29] Siegler, R. S., & Stern, E. (1998). Conscious and unconscious strategy discoveries: A microgenetic analysis. Journal of Experimental Psychology: General, 127(4), 377–397. DOI: 10.1037/0096-3445.127.4.377 9857493

[30] Sommerauer, G., Graß, K. H., Grabner, R. H., & Vogel, S. E. (2020). The semantic control network mediates the relationship between symbolic numerical order processing and arithmetic performance in children. Neuropsychologia, 141. DOI: 10.1016/j.neuropsychologia.2020.107405 32087204

[31] Steiger, J. H. (1980). Tests for comparing elements of a correlation matrix. Psychological Bulletin, 87(2), 245–251. DOI: 10.1037/0033-2909.87.2.245

[32] Torbeyns, J., Peters, G., De Smedt, B., Ghesquière, P., Verschaffel, L. (2016). Children’s understanding of the addition/subtraction complement principle. British Journal of Educational Psychology, 86, 382–396. DOI: 10.1111/bjep.12113 26990792

[33] Torbeyns, J., Verschaffel, L. (2016). Mental computation or standard algorithm? Children’s strategy choices on multi-digit subtractions. European Journal of Psychology of Education, 31, 99–116. DOI: 10.1007/s10212-015-0255-8

[34] Turconi, E., Campbell, J. I. D., & Seron, X. (2006). Numerical order and quantity processing in number comparison. Cognition, 98(3), 273–285. DOI: 10.1016/j.cognition.2004.12.002 16399265

[35] Turconi, E., Jemel, B., Rossion, B., & Seron, X. (2004). Electrophysiological evidence for differential processing of numerical quantity and order in humans. Cognitive Brain Research, 21(1), 22–38. DOI: 10.1016/j.cogbrainres.2004.05.003 15325410

[36] van’t Noordende, J. E., van Hoogmoed, A. H., Schot, W. D., & Kroesbergen, E. H. (2016). Number line estimation strategies in children with mathematical learning difficulties measured by eye tracking. Psychological Research, 80(3), 368–378. DOI: 10.1007/s00426-015-0736-z 26708497 PMC4826415

[37] Vogel, S. E., Haigh, T., Sommerauer, G., Spindler, M., Brumner, C., Lyons, I. M., & Grabner, R. (2017). Processing the order of symbolic numbers: A reliable and unique predictor of arithmetic fluency. Journal of Numerical Cognition, 3(2), 288–308. DOI: 10.5964/jnc.v3i2.55

[38] Vogel, S. E., Koren, N., Falb, S., Haselwander, M., Spradley, A., Schadenbauer, P., Tanzmeister, S., & Grabner, R. H. (2019). Automatic and intentional processing of numerical order and its relationship to arithmetic performance. Acta Psychologica, 193, 30–41. DOI: 10.1016/j.actpsy.2018.12.001 30584972

[39] Vogel, S. E., Remark, A., & Ansari, D. (2014). Differential processing of symbolic numerical magnitude and order in first- grade children. Journal of Experimental Child Psychology, 129, 26–39. DOI: 10.1016/j.jecp.2014.07.010 25240153

