# Supplementary material for: Symbolic Number Ordering and its Underlying Strategies Examined Through Self-Reports
Source: J Cogn. 2021 Apr 12;4(1):25. doi: 10.5334/joc.157 (PMC8051157; doi:10.5334/joc.157)
Supplement: Appendix 1. — Stimulus list. [file joc-4-1-157-s1.pdf]

Appendix 1. Stimulus list

Triplet			Condition	Direction Non-ordered	Distance	Correctness
1	2	3	AS	Ascending	1	Correct
3	2	1	DS	Descending	1	Correct
2	3	4	AS	Ascending	1	Correct
4	3	2	DS	Descending	1	Correct
3	4	5	AS	Ascending	1	Correct
5	4	3	DS	Descending	1	Correct
4	5	6	AS	Ascending	1	Correct
6	5	4	DS	Descending	1	Correct
5	6	7	AS	Ascending	1	Correct
7	6	5	DS	Descending	1	Correct
6	7	8	AS	Ascending	1	Correct
8	7	6	DS	Descending	1	Correct
7	8	9	AS	Ascending	1	Correct
9	8	7	DS	Descending	1	Correct
2	1	3	NS	Non-ordered	1	Incorrect
1	3	2	NS	Non-ordered	1	Incorrect
4	2	3	NS	Non-ordered	1	Incorrect
2	4	3	NS	Non-ordered	1	Incorrect
5	3	4	NS	Non-ordered	1	Incorrect
3	5	4	NS	Non-ordered	1	Incorrect
4	6	5	NS	Non-ordered	1	Incorrect
5	4	6	NS	Non-ordered	1	Incorrect
6	5	7	NS	Non-ordered	1	Incorrect
5	7	6	NS	Non-ordered	1	Incorrect
8	6	7	NS	Non-ordered	1	Incorrect
7	8	6	NS	Non-ordered	1	Incorrect
7	9	8	NS	Non-ordered	1	Incorrect
8	7	9	NS	Non-ordered	1	Incorrect
5	3	1	DL	Descending	2	Correct
2	4	6	AN	Ascending	2	Correct
6	4	2	DN	Descending	2	Correct
3	5	7	AL	Ascending	2	Correct
4	6	8	AL	Ascending	2	Correct
8	6	4	DL	Descending	2	Correct
5	7	9	AL	Ascending	2	Correct
1	4	7	AL	Ascending	3	Correct
7	4	1	DL	Descending	3	Correct
2	5	8	AL	Ascending	3	Correct
8	5	2	DL	Descending	3	Correct
9	6	3	DL	Descending	3	Correct
1	5	9	AL	Ascending	4	Correct
9	5	1	DL	Descending	4	Correct
5	1	3	NL	Non-ordered	2	Incorrect

4	2	6	NL	Non-ordered	2	Incorrect
3	7	5	NL	Non-ordered	2	Incorrect
6	8	4	NL	Non-ordered	2	Incorrect
4	8	6	NL	Non-ordered	2	Incorrect
5	9	7	NL	Non-ordered	2	Incorrect
7	9	5	NL	Non-ordered	2	Incorrect
4	1	7	NL	Non-ordered	3	Incorrect
8	2	5	NL	Non-ordered	3	Incorrect
5	8	2	NL	Non-ordered	3	Incorrect
6	9	3	NL	Non-ordered	3	Incorrect
3	9	6	NL	Non-ordered	3	Incorrect
1	9	5	NL	Non-ordered	4	Incorrect
5	1	9	NL	Non-ordered	4	Incorrect

Conditions: AS – ascending small, DS – descending small, NS – non-ordered small, AL – ascending large, DL – descending large, NL – non-ordered large
